# Adolescent traumatic brain injuries: Onset, mechanism and links with current academic performance and physical injuries

**DOI:** 10.1371/journal.pone.0229489

**Published:** 2020-03-12

**Authors:** Gabriela Ilie, Michelle Trenholm, Angela Boak, Robert E. Mann, Edward M. Adlaf, Mark Asbridge, Hayley Hamilton, Jürgen Rehm, Robert Rutledge, Michael D. Cusiman

**Affiliations:** 1 Department of Community Health and Epidemiology, Dalhousie University, Halifax, Canada; 2 Faculty of Medicine, Dalhousie University, Halifax, Canada; 3 Institute for Mental Health Policy Research, Centre for Addiction and Mental Health, Toronto, Canada; 4 Dalla Lana School of Public Health, University of Toronto, Toronto, Canada; 5 Department of Radiation Oncology, Dalhousie University, Halifax, Canada; 6 Division of Neurosurgery, University of Toronto, Toronto, Canada; University of Bern, SWITZERLAND

## Abstract

**Background:**

Traumatic brain injuries (TBIs) in adolescence are associated with adverse outcomes, but whether the timing of the onset of TBIs leads to greater deficits has not been determined. We evaluate the relationship between the first and most recent TBI, and current academic performance and medically treated physical injuries.

**Methods:**

Data were derived from the 2015 Ontario Student Drug Use and Health Survey (OSDUHS) administered to adolescents in grades 7 to 12 (ages 12 to 18). TBI was defined as a head injury that resulted in loss of conscious for at least five minutes or at least one overnight hospitalization.

**Results:**

One in five students reported having had a history of TBI in their lifetime and were more prevalent in males. Odds ratios were 2 times higher for males to have had their first (or only) and most recent TBI in grades 5 to 8, compared to females. Sports-related TBIs accounted for 41.1% of all TBIs. Hockey related TBIs were more frequent compared to soccer related TBIs. Reports of history of TBI was associated with lower academic performance and more physical injuries. First or only TBI occurring in grades 9–12 (occurring on average between 14 to 19 years of age) had higher significant odds of poorer academic performance than TBIs occurring in earlier grades (younger ages than 14 years old). Students who reported more visits for medical treatment of physical injuries in the past year had higher odds to report a history of TBIs in higher school grades.

**Conclusions:**

Adverse physical and academic outcomes among young TBI survivors are associated with the onset and frequency of history of lifetime TBI. Prevention efforts to minimize TBIs during youth is critical.

## Introduction

Traumatic brain injuries (TBI) among adolescents have been identified as a significant global public health issue in the past 15 years.[1–4] Recent reports show that 15 to 19 year-olds have some of the highest emergency department visits, hospitalizations, and deaths related to TBIs compared to any other age group.[5–10] Results from both Canada[2,11–13] and US[4] show that about 22% or 20% of adolescents sustain a TBI in their lifetime, respectively, and as many as 5.5% have multiple injuries.[4] These injuries co-occur with alcohol use, binge drinking, use of energy drinks mixed with alcohol, hazardous drinking, other substance use, low school grades, mental health issues, violent and anti-social behaviors.[2,11–15] TBIs are particularly common among males and during sport activities.[2,13]

Trauma to the head during adolescence, while the brain is still developing, has been shown to lead to poor quality of life indicators in the areas of cognitive, emotional, behavioural, and social development.[4,5,11,12,14–16] Adolescents who report having had a history of TBI also report having had more physical injuries than their peer who do not have a TBI.[14,17] Among female adolescent TBI survivors, those aged 11 to 13 years report more medically treated physical injuries than older female survivors while the reverse has been found for males.[14] TBIs in adolescence extend from associations to increased number of physical injuries to increased risk behaviours, suicidality and substance use.[11,12,18–22] TBIs can cause physical impairments such as loss of balance, and visual and auditory problems that can, subsequently, increase susceptibility to subsequent TBIs, falls and physical injuries.[14,15,23]

However, little is known about the temporal spacing of TBIs and any differences among associated adverse outcomes based on when the injury occured.[15,24] There have been mixed reports on whether an earlier or later onset of the first TBI during youth results in worse outcomes. Some evidence in the literature indicates that TBIs incurred during early childhood (before 2 to 5 years old) and during early adolescence (around 11–13 years of age) lead to worse short and long term quality of life outcomes.[5,24–29] Brain trauma in early childhood is associated with more generalized deficits on brain function as opposed to a single cognitive or social deficit.[25,27] TBIs first occurring between early childhood and adolescence have been associated with the least adverse outcomes;[26,27] although, it has been argued that TBIs incurred during this time may not result in deficits until later higher level cognitive and social skills would be expected to be developed.[27,29] TBIs in adolescence showed adverse outcomes in adults in a large population-based Swedish study. Yet another study reported that among adults who had a TBI before the age of 25, as age of TBI onset increased worse psychiatric health, greater need for welfare or disability pension, lower levels of education, and more pre-mature mortality were found.[30]

Few studies have examined the relationship between TBI onset or the timing of the most recent injury and multiple injuries.[24] This is an important consideration given that understanding these associations is important for managing emerging negative health outcomes in this population, during, what is believed to be a most critical and sensitive developmental period.[27] In general, most studies find that greater deficits are observed acutely, right after a TBI’s onset.[24] TBIs that occurred within the past 12 months have been associated with worse academic performance among adolescents, compared to TBIs that occurred more than 12 months previously.[11] Research also found that multiple TBIs may also be associated with worse outcomes, especially if the TBI occurred before the brain has had time to heal from the previous TBI.[5,23,31]

To our knowledge, no large-scale epidemiological surveys of North American adolescents have previously assessed when an adolescent’s first (or only) and most recent TBI occurred, the mechanism of injury associated with the most recent TBI, and how the grade in which the first or last TBI occurred predicts current academic performance and physical injuries requiring medical treatment within the previous 12 months. To address this gap in the literature we utilized data from the 2015 Ontario Student Drug Use and Health Survey (OSDUHS) of student in grades 7 to 12. Additionally, we describe the prevalence of TBIs and assess key factors (i.e., participation in sports) known to be associated with TBIs within a large representative sample of Ontario adolescents.

## Methods

Data was based on a sample of 10,426 students in grades 7 to 12 who participated in the 2015 cycle of the Ontario Student Drug Use and Health Survey (OSDUHS), a biennially repeated cross-sectional probability survey of Ontario students enrolled in public funded schools in the province.[32] This survey collects data on a range of health and health-related issues, including drug use, well-being, and risk behaviours. The survey emphasizes complete anonymity and privacy, to encourage truthful responses from students on sensitive matters. In 2015, students were recruited from 43 school boards, 220 schools and 750 classes dispersed province wide. In Ontario, 7th and 8th graders can be enrolled in elementary schools (Junior Kindergarden–Grade 8), middle or senior public schools (Grade 6 to Grade 8), or junior high schools (Grade 7 to Grade 9). Publicly funded schools represented by four school systems in Ontario–English and French language schools in the public and Catholic school sectors–were eligible to participate. Schools excluded as being out-of-scope were private schools, schools on First Nations reserves, on Canadian Forces Bases, and schools in geographically inaccessible northern areas.

Schools excluded from sampling were private, military, and institutional schools. With these exclusions, our sample captures 92% of all Ontario children and adolescents aged 12 to 18.[32]

Of the 17671 enrolled in the classes randomly selected for the administration of the survey, 10426 completed self-administered, anonymous pen-and-paper questionnaires in their classrooms (considered “completed”) between November 2014 and June 2015. This resulted in a participation rate of 59% for students. This is comparable to other Ontario surveys using active consent procedures (required by the vast majority of school boards in Ontario).[33]

In total, 349 schools (273 initial selections plus 76 replacements) were invited to participate. Of these, **220 schools** (103 elementary/middle–of which four were French language–and 117 secondary–of which three were French language) from 43 school boards participated in the survey, resulting in a school participation rate of 63%. The most common reasons given by nonparticipating schools were that they were too busy, or that they had already committed to other research projects. Each school that was unable to participate was replaced with a randomly selected school from the same stratum and with similar school size in order to maintain representativeness. A total of **750 classes** met the class inclusion criteria and participated in the survey (286 from elementary/middle schools, 464 from secondary schools). The class participation rate was 88%. We must note that 90 (12%) classes were not randomly selected. Rather, these classes were convenient same-grade replacements, typically identified by principals, for classes that were originally selected but declined to participate for logistical reasons.

A comparison between high (≥ 70%) and low responding classes showed no evidence of nonresponse bias for a number of health-related behaviours, including TBI. Students completed one of two questionnaires (Form A or Form B) alternately distributed (i.e., A, B, A) within each class. The TBI and academic performance items were asked in both forms; the medically treated physical injuries in the last 12 months item was only asked in Form B. Detailed description of the sampling design and survey procedures, questions and their validation, validity of self-reports, nonresponse bias, and limitations is web-available.[32] The study was approved by the Research Ethics Committees of the Centre for Addiction and Mental Health (CAMH), St. Michael’s Hospital (SMH), participating Ontario Public and Catholic school boards, and York University, which administered the survey. The survey was conducted according to the principles of the Declaration of Helsinki. All participants provided their assent in addition to parentally signed consent for those aged under 18.[32]

## Measures

### History of traumatic brain injury (TBI)

For this study, a TBI was defined as a hit to the head that resulted in at least five minutes of lost consciousness, or at least one overnight hospitalization.[32] This definition of TBI is used by several diagnostic classification systems including DSM-IV and has previously been used in adolescent and adult studies.[18,34–36] Students were asked if they ever had a head injury like this in their lifetime. Responses were (1) never had a head injury like this in my life, (2) had a head injury like this, but not in the last 12 months, and (3) had a head injury like this in the past 12 months and were coded the same.

### Frequency of TBI

Students were asked how many TBIs they had in their lifetime. Responses were coded as (1) never had a TBI, (2) one, (3) two, (4) between three to five, and (5) for six or more past TBIs.

### School grade at first (or only) TBI

Students were asked to provide the school grade in which they were in at the time they had their first (or only) TBI. Responses were coded (1) never had a TBI, (2) grade 4 or before, (3) in grades 5 to 8, and (4) in grades 9 to 12.

### School grade at most recent TBI

Students were also asked in which school grade they were at the time they incurred their most recent TBI. Responses were coded (1) never had a TBI, (2) did not have more than one TBI, (3) grade 4 or before, (4) in grades 5 to 8, and (5) in grades 9 to 12.

### Mechanism of TBI

Students were asked to provide the cause of their most recent TBI. Responses were coded (1) motor vehicle accident, (2) other vehicle accident, (3) bicycle accident, (4) playing hockey, (5) playing soccer, (6) playing another team sport, (7) other sports injury, (8) fell down by accident, (9) was in a fight with someone, (10) bullied (pushed) by someone, (11) someone threw an object at them on purpose, (12) an object hit them by accident, (13) other cause not listed, and (14) multiple answers.

### Current academic performance

Students were asked on average, what grades they currently receive in school. Responses 1 (90% to 100%) and 2 (80% to 89%) were coded as (1); response 3 (70% to 79%) was coded as (2); and response 4 (60% to 69%), 5 (50% to 59%) and 6 (below 50%) were coded as (3).

### Number of visits to medically treat any physical injury in the last 12 months

Students were asked how many times they had sought medical treatment in the last 12 months for any physical injuries. Responses were (1) was not treated for an injury in the last 12 months, (2) once, (3) twice, (4) three times, and (5) four or more times, and were coded the same.

Sex was assessed and was coded as 0 (female) or 1 (male). School grade was coded (1) grade 7, (2) grade 8, (3) grade 9, (4) grade 10, (5) grade 11, and (6) grade 12.

### Statistical analyses

Data derived from complex surveys using stratification and clustering do not meet the assumption of independent observations and thus underestimate variances, overstating significance levels which may result in false positive inferences. We therefore employed design-based estimation methods to accommodate these sampling methods. Our subsample analysis utilized a complex sample design with 21 region-by-school level strata, 220 schools (primary sampling unit clusters), and 750 classes (secondary sampling unit clusters).[32] To account for the complex sampling the OSDUHS utilized (stratification, clustering and unequal sampling) Taylor Series Linearization in the Complex Sample Module of SPSS version 23 (SPSS Inc., 2015) was used for statistical analyses. Weighted estimates by sex and grade distribution were determined for the outcomes of interest. Multinomial logistic regression was used to compute the odds ratios for sex, current school performance, the number of visits to medically treat any physical injury in the last 12 months, and frequency of TBI by school grade at first (or only) TBI and school grade at most recent TBI. The level of statistical significance was *p* < 0.05 (two tailed). The results are based on ‘valid’ responses (n’s); missing data (i.e. ‘don’t know’ responses and refusals) were excluded. Listwise deletion reduced the estimation sample from 10,426 to 10,270, 10,267 and 10,261 for history of TBI, school grade at first (or only) TBI and school grade at most recent TBI, respectively.

## Results

Table 1 presents demographic characteristics, the number of visits to medically treat any physical injuries in the last 12 months, and frequency of TBI for the history of TBI categorization. An estimated 14% (95% CI:12.9,15.1) reported a history of TBI in their lifetime (but not in the past 12 months), 4.9% (95% CI:4.1,5.8) reported a TBI in the past 12 months, and 81.1% (95% CI:79.7,82.4) reported they never had a TBI in their lifetime. History of TBI did not differ significantly among grades but were more prevalent among males compared to females for both recent and lifetime TBI. One or two TBIs were most frequently reported among male (83.5% and 74.8%, respectively) or female (82.2% and 71.1%, respectively) students who reported a history TBI in their lifetime but not in the past 12 months. Three to five TBIs were just as frequent among students who reported lifetime (excluding past 12 months) TBI (females 45.7%, males 52.5%) than those who reported a history of TBI in the past 12 months (females 54.3%, males 47.5%). For both males and females, history of six or more TBI was more frequent among students who reported a history of TBI in the past 12 months (females 81.5%, males 71.4%) than those who reported lifetime TBIs (excluding the past 12 months). The frequency of the number of visits to medically treat any physical injuries in the last 12 months for students in grades 7 through 12, showed an overall significant increasing pattern for both males and females who reported at least one TBI in the past 12 months. History of TBIs did not differ statistically between grades. The four most frequent mechanisms of TBI injury in the past 12 months were injury due to falling-down by accident (19.5%; 95% CI:17.2,21.9), other causes not surveyed (16.7%; 95% CI:14.8,18.9), playing hockey (14.2; 95% CI:11.7,17.2), and playing another team sport than hockey (14.6; 95% CI: 12.1,17.3). Sports related injuries accounted for 41.4% of all reported mechanisms of injury in past 12 months TBIs. Hockey related TBIs were more frequent compared to soccer related TBIs.

**Table 1:** Weighted estimates of TBI by demographic characteristics, TBI frequency, and the number of visits to medically treat physical injuries in the last 12 months for Ontario 7^th^ to 12^th^ graders, 2015 OSDUHS (n = 10270).

	Sample characteristics	Never	Lifetime^a^	Past 12 months
	% (95% CI)	% (95% CI)	% (95% CI)	% (95% CI)
	n = 10270	n = 8232	n = 1500	n = 538
Sex	*F* (2,395) = 28.33*			
Female	48.3 (45.8,50.9)	85.6 (84.0,87.0)	10.5 (9.3,11.9)	3.9 (3.1,4.9)
*Number of visits to medically treat physical injuries in the last 12 months*	*F* (8,180) = 11.70**			
None		91.3 (89.0,93.1)	6.9 (5.3,9.0)	1.8 (1.0,3.3)
1 time		83.3 (78.1,87.5)	12.3 (9.1,16.4)	4.4 (2.7,7.1)
2 times		72.5 (63.4,80.1)	18.9 (13.0,26.6)	8.6 (4.9,14.8)
3 times		52.1 (36.8,67.0)	31.9 (17.9,50.1)	16. 0 (6.9,32.8)
4 or more times		50.2 (38.3,62.0)	24.9 (14.8,38.9)	24.9 (14.8,38.8)
*TBI frequency*	*F* (7,1283) = 274.95*			
1			82. 2 (77.0,86.5)	17.8 (13.5,23.0)
2			71.1 (60.7,79.7)	28.9 (20.3,39.3)
3 to 5			45.7 (27.6,65.0)	54.3 (35.0,72.4)
6 or more			18.5 (5.4,47.4)	81.5 (52.6,94.6)
Male	51.7 (49.1,54.2)	76.9 (74.9,78.9)	17.2 (15.7,18.8)	5.9 (4.8,7.2)
*Number of visits to medically treat physical injuries in the last 12 months*	*F* (8,180) = 8.93**			
None		82.4 (79.0,85.3)	15.4 (12.7,18.7)	2.2 (1.4,3.4)
1 time		72.4 (64.8,78.8)	19.4 (14.7,25.2)	8.2 (4.9,13.5)
2 times		61.7 (50.1,72.1)	23.4 (16.1,32.7)	14.9 (8.9,23.9)
3 times		68.8 (55.3,79.7)	19.5 (11.8,30.5)	11.7 (6.3,20.7)
4 or more times		56.1 (42.5,68.8)	19.8 (11.4,32.2)	24.1 (13.5,39.3)
*TBI frequency*	*F* (6,1130) = 285.27*			
1			83.5 (78.5,87.5)	16.5 (12.5,21.5)
2			74.8 (66.4,81.7)	25.2 (18.3,33.6)
3 to 5			52.5 (40.4,64.4)	47.5 (35.6,59.6)
6 or more			28.6 (13.4,50.9)	71.4 (49.1,86.6)
Grade	*F* (1,7) = 28.61			
7	12.9 (11.6,14.3)	79.0 (75.8,81.8)	14.9 (12.5,17.7)	6.1 (4.6.8.0)
8	13.6 (12.5,14.7)	83.2 (78.7,86.8)	12.9 (9.6,17.1)	4.0 (2.7,5.7)
9	16.1 (15.1,17.1)	81.7 (79.2,84.0)	14.1 (12.0,16.4)	4.2 (3.0,5.9)
10	16.4 (15.2,17.7)	79.7 (76.9,82.3)	15.5 (13.6,17.7)	4.7 (3.4,6.7)
11	17.3 (16.5,18.1)	79.2 (76.3,81.9)	15.5 (13.2,18.2)	5.3 (3.7,7.4)
12	23.8 (21.9,25.8)	83.0 (79.3,86.2)	11.8 (9.9,14.1)	5.2 (3.4,7.9)

^a^ Lifetime, excluding past 12 months

* *p* < 0.05

** *p* < 0.001

Multinomial logistic regression (adjusted for the complexity of the design) revealed that sex, current school marks, and the number of visits to medically treat any physical injuries in the last 12 months were significant predictors of school grade at first injury, *F* (3,197) = 23.64, *p* < 0.001, *F* (15,185) = 4.88, *p* < 0.001, and *F* (12,176) = 9.82, *p* < 0.001, respectively (Table 2). Odds ratios were double for males compared to females to report the occurrence of their first TBI in grade 8 or before, but were similar between sexes for TBIs that were reported to have occurred between grades 9 to 12. Among students who reported the occurrence of their first TBI between grades 5 to 8, odds ratios were almost three times greater (2.73, 95% CI:1.92,3.89) for reports of current school marks between below 69%. Odds ratios were 3.22 (95% CI: 1.78,5.84) times greater to report poorer school performance (current school marks below 69%) among students who reported their first TBI occurrence between grades 9 to 12. Odds ratios for one or more visits for medical treatment of any physical injuries in the last 12 months were statistically significant and highest among students who reported the occurrence of the first TBI in higher grades (grades 9 to 12).

**Table 2:** Weighted estimates and multinomial logistic regression analyses for grade at first (or only) TBI by sex, current academic performance, and the number of visits to medically treat any physical injuries in the last 12 months for Ontario 7^th^–12^th^ graders, 2015 OSDUHS (n = 10267).

	Total	Never	Grade 4 or before	Grade 5 to 8	Grade 9 to 12
		OR (95% CI)	OR (95% CI)	OR (95% CI)	OR (95% CI)
		% (95% CI)	% (95% CI)	% (95% CI)	% (95% CI)
	n = 10267	n = 8232 81.1 (79.6,82.4)	n = 898 7.9 (7.1,8.8)	n = 880 8.0 (7.2,8.8)	n = 257 3.1 (2.5,3.7)
Sex		*F* (3,197) = 23.64***			
*Females*		1 [Reference]	1 [Reference]	1 [Reference]	1 [Reference]
	48.4 (45.8,50.9)	85.5 (84.0,86.9)	5.9 (5.0,6.9)	5.8 (5.0,6.8)	2.8 (2.1,3.7)
*Males*		1 [Reference]	1.84 (1.51,2.24)***	1.90 (1.51,2.39)***	1.34 (0.92,1.96)
	51.6 (49.1,54.2)	76.9 (74.8,78.9)	9.8 (8.6,11.0)	10.0 (8.7,11.4)	3.4 (2.6,4.3)
Current school marks^a^		*F* (15,185) = 4.88***			
*80–100%*		1 [Reference]	1 [Reference]	1 [Reference]	1 [Reference]
	56.3 (54.1,58.5)	84.7 (83.2,86.0)	6.7 (5.8,7.9)	6.2 (5.4,7.0)	2.4 (1.9,3.0)
*70–79%*		1 [Reference]	1.44 (1.10,1.90)	1.57 (1.26,1.94)*	1.54 (1.06,2.26)
	36.1 (34.4,37.9)	77.6 (75.0,80.1)	9.5 (7.9,11.3)	9.4 (8.0,11.1)	3.5 (2.6,4.7)
*69% or below*		1 [Reference]	1.43 (0.97,2.11)	2.73 (1.92,3.89)***	3.22 (1.78,5.84)***
	7.6 (6.7,8.7)	69.6 (63.9,74.8)	8.7 (6.4,11.6)	15.1 (11.6,19.6)	6.6 (4.0,10.6)
Number of visits to medically treat physical injuries in the last 12 months^a^		*F* (12,176) = 9.82***			
*None*		1 [Reference]	1 [Reference]	1 [Reference]	1 [Reference]
	56.4 (53.7,59.0)	86.8 (84.8,88.6)	7.3 (6.0,8.8)	4.5 (3.3,6.0)	1.4 (0.9,2.4)
*1*		1 [Reference]	1.30 (0.90,1.89)	2.20 (1.38,3.51)**	3.78 (2.01,7.11)***
	22.6 (20.8,24.6)	77.5 (73.6,81.1)	8.6 (6.4,11.5)	9.0 (6.6,12.1)	4.9 (3.2,7.4)
*2*		1 [Reference]	2.30 (1.28,4.14)*	4.52 (2.86,7.14)*	5.41 (2.51,11.65)***
	11.3 (9.7,13.2)	65.9 (56.7,74.0)	12.8 (8.4,19.0)	15.4 (11.3,20.8)	5.9 (3.4,10.0)
*3*		1 [Reference]	3.25 (1.52,6.96)*	3.36 (1.82,6.21)***	11.63 (4.05,33.37)***
	5.3 (4.3,6.5)	60.8 (49.3,71.3)	16.8 (9.3,28.2)	10.7 (6.6,16.8)	11.7 (5.3,24.0)
*4 or more*		1 [Reference]	3.12 (1.66,5.86)***	8.80 (5.18,14.96)***	9.45 (3.84,23.27)***
	4.4 (3.6,5.4)	54.1 (45.5,62.4)	13.7 (8.2,22.0)	23.8 (17.3,31.8)	8.4 (4.2,15.9)

*** *p* < 0.001

** *p* < 0.01

* *p* < 0.05

^a^ controlling for the complexity of the design

Multinomial logistic regression (adjusted for the complexity of the design) revealed that sex, current school marks, and the number of visits to medically treat any physical injuries in the last 12 months were significant predictors of school grade at most recent injury, *F* (4,196) = 15.43, *p* < 0.001, *F* (8,192) = 6.52, *p* < 0.001, and *F* (16,172) = 10.81, *p* < 0.001, respectively (Table 3). Odds ratios were double for males compared to females to report the occurrence of their most recent TBI in grades 5 to 8. For students who had their most recent TBI between grades 5 to 8, or between grades 9 to 12, the odds ratios were 2.23 (95% CI:1.34,3.37) and 3.67 (95% CI:2.46,5.48) times greater for reporting poor school performance (current school marks 69% or below). Among students who reported one TBI in their lifetime, the odds ratios were 2.12 (95% CI:1.53,2.95) times greater for reporting current school marks 69% or below. Odds ratios were highest for increased number of visits to seek medical treatment for any physical injuries in the past 12 months for students who reported their most recent TBI between grades 9 to 12 compared to other grades. Among students who reported one TBI in their lifetime odds ratios for one or two visits to see a doctor for medical treatment of any physical injuries in the last 12 months were double (2.04 and 2.19, respectively).

**Table 3:** Weighted estimates and multinomial logistic regression analyses for grade at most recent (if more than one) TBI by sex, current academic performance, and the number of visits to medically treat any physical injuries in the last 12 months for Ontario 7^th^–12^th^ graders, 2015 OSDUHS (n = 10261).

	Total	Never	Only one TBI	Grade 4 or before	Grade 5 to 8	Grade 9 to 12
		OR (95% CI)	OR (95% CI)	OR (95% CI)	OR (95% CI)	OR (95% CI)
		% (95% CI)	% (95% CI)	% (95% CI)	% (95% CI)	% (95% CI)
	n = 10261	n = 8283 81.6 (80.3,82.9)	n = 859 8.0 (7.3,8.8)	n = 262 2.5 (2.0,3.0)	n = 533 4.0 (3.5,4.6)	n = 324 3.8 (3.0,4.8)
Sex	*F* (4,196) = 15.43***					
*Females*		1 [Reference]	1 [Reference]	1 [Reference]	1 [Reference]	1 [Reference]
	48.4 (45.8,50.9)	85.7 (84.2,87.1)	5.7 (4.9,6.8)	2.3 (1.7,3.1)	2.8 (2.3,3.4)	3.5 (2.7,4.6)
*Males*		1 [Reference]	1.96 (1.57,2.44***	1.28 (0.89,1.85)	2.10 (1.58,2.79)***	1.27 (0.96,1.70)
	51.6 (49.1,54.2)	77.8 (75.9,79.7)	10.2 (9.1,11.4)	2.6 (2.1,3.3)	5.3 (4.4,6.3)	4.1 (3.1,5.4)
Current school marks^a^	*F* (8,192) = 6.52***					
*80–100%*		1 [Reference]	1 [Reference]	1 [Reference]	1 [Reference]	1 [Reference]
	56.6(54.4,58.8)	85.2 (83.7,86.5)	6.8 (5.8,7.8)	2.2 (1.7,2.8)	3.2 (2.6,3.9)	2.7 (2.0,3.6)
*70–79%*		1 [Reference]	1.37 (1.09,1.72)	1.54 (0.98,2.43)	1.59 (1.15,2.21)*	1.79 (1.16,2.77)*
	35.9 (34.2,37.7)	78.3 (75.8,80.5)	9.1 (7.9,10.5)	3.1 (2.3,4.4)	5.0 (4.0,6.2)	4.5 (3.2,6.3)
*69% or below*		1 [Reference]	2.12 (1.53,2.95)**	0.85 (0.41,1.76)	2.23 (1.34,3.37)**	3.67 (2.46,5.48)***
	7.5 (6.6,8.5)	70.7 (65.7,75.2)	13.1 (10.2,16.7)	1.6 (0.8,3.0)	6.2 (4.5,8.7)	8.4 (5.6,12.3)
Number of visits to medically treat physical injuries in the last 12 months^a^	*F* (16,172) = 10.81***					
*None*		1 [Reference]	1 [Reference]	1 [Reference]	1 [Reference]	1 [Reference]
	56.4 (53.7,59.0)	87.8 (85.9,89.4)	5.9 (4.8,7.1)	2.4 (1.6,3.4)	2.6 (1.8,3.6)	1.4 (0.9,2.2)
*1*		1 [Reference]	2.04 (1.39,2.97**	1.23 (0.51,2.96)	1.77 (1.02,3.07)	3.44 (1.80,6.60)**
	22.6 (20.8,24.6)	78.1 (74.1,81.6)	10.9 (8.5,13.8)	2.6 (1.3,5.0)	4.1 (2.6,6.3)	4.3 (2.7,6.9)
*2 times*		1 [Reference]	2.19 (1.27,3.77)**	1.95 (0.79,4.81)	4.30 (2.37,7.81)***	9.24 (4.78,17.87)***
	11.3 (9.6,13.2)	67.7 (59.4,75.1)	10.1 (6.4,15.4)	3.6 (1.7,7.2)	8.6 (5.6,12.9)	10.1 (6.4,15.5)
*3 times*		1 [Reference]	1.69 (0.90,3.18)	4.18 (1.05,16.56)	3.57 (1.79,7.15)***	18.47 (8.99,37.98)***
	5.3 (4.3,6.4)	61.2 (49.7,71.7)	7.1 (4.3,11.4)	6.9 (2.2,19.7)	6.5 (3.6,11.3)	18.3 (10.1,30.6)
*4 or more times*		1 [Reference]	1.92 (0.77,4.80)	3.21 (1.42,7.27)**	9.97 (5.48,18.13)***	23.10 (12.01,44.42)***
	4.4 (3.6,5.5)	53.6 (44.7,62.2)	6.8 (2.9,15.0)	4.6 (2.0,9.9)	15.2 (10.2,22.1)	19.8 (13.2,28.8)

*** *p* < 0.001

** *p* < 0.01

* *p* < 0.05

^a^ controlling for the complexity of the design.

## Discussion

Data from the 2015 OSDUHS showed that the prevalence of TBIs in Ontario adolescents, 18.9%, is comparable to the prevalence reported 4 years earlier among this population.[2] The prevalence of lifetime TBI, excluding the last 12 months, for the last three cycles of OSDUHS was 14.6%, 16.3% and 14.0% for 2011, 2013 and 2015, respectively.[2,13] The prevalence of TBIs in the last 12 months was 5.6%, 6.0% and 4.9% for 2011, 2013 and 2015, respectively.[2,13] taken together (lifetime TBI except past 12 months, and TBIs which occurred in the past 12 months, 20.2%, 22.3%, and 18.9%, respectively) these prevalence estimates are comparable to those of other population-based studies and that they remain a major public health issue. A national 2016 cross-sectional survey study of U.S. adolescents in grades 8 to 12 revealed that approximately 19.5% of the respondents reported at least one diagnosed concussion in their lifetime.[4]

The grade in which adolescents’ first (or only) and most recent TBI occurred was significantly associated with sex, poor academic performance, and the number of visits for medical treatment of physical injuries in the last 12 months. History of lifetime TBI was more prevalent in males compared to females, which corroborates the results of previous studies.[5,37–39] The odds ratio for having had the most recent TBI (or only TBI) occurring between grades 5 to 8 and grade 4 or earlier were almost double for male adolescents compared to female adolescents (1.90 and 1.84, respectively) suggesting earlier onset TBI for boys compared to girls. This data indicates that males are more likely to have their first TBI injury before age 14. Male adolescents had almost double the odds than female adolescents to have had only one TBI in their lifetime. This finding is consistent with the literature.[5,14,40] Male adolescents in this sample also had more than double the odds of sustaining their most recent TBI between grades 5 to 8 compared to female adolescents. To our knowledge this is the first report on this type of association in the adolescent population.

Here we report no differences between reported history of TBIs among various school grades compared to the 2011 OSDUHS cycle data where students in grade 7 and 8 were 2 to 3 times more likely than students in older grades to report a TBI within the last 12 months.[2] Our results indicate that among the students who reported more than one TBIs in their lifetime, they also had higher odds of having incurred a TBI in the past year. This observation is consistent with previous research suggesting that the risk for a TBI increases with the frequency of TBIs.[15,23]

Depending on the time of occurrence, poorer current academic performance (69% or lower average school marks) was associated with, both, the first (or only) TBI and the most recent TBI, when occurring between grades 5 to 12. Reports of TBI occurrence (either first or most recent) before grade 5 or age 10 was not statistically significantly associated with poor self-reported academic performance in this sample. Reporting a history of one TBI during lifetime was associated with more than double the odds of poor school performance (school grades 69% or below). Thus, results indicate that, depending on the time of occurrence, even one history of TBI event can have an adverse association with academic performance during elementary and high-school years.

Particularly, students had 3 times higher odds for reporting poorer average school marks (69% or below) if their first (or only) TBI occurred in grades 5 to 12. Students who had current average school marks of 69% or below had greater odds of having their first (or only) TBI in grades 9 to 12 compared to students who had their first TBI in other grades. These results suggest that first or only TBIs occurring in grades 9 to 12 (between the ages of 14 to 18) have a greater impact on current academic performance than TBIs occurring in earlier grades (before 14 years of age). Prevention efforts to minimize these injuries during youth, particularly during adolescent years, is warranted.

This data expands results from previous administrations of the OSDUHS showing that a history of TBI is associated with poor academic performance, especially for adolescents who reported a TBI in the past 12 months.[2,14,15] Further studies should assess whether more recent TBIs acutely (short-term) or chronically (long-term) impact quality of life outcomes (e.g. mental health) in this population. Here we report that self-reported TBIs occurring in grades 4 and before (below 10 years of age) were not associated with academic performance. However, it is important to be cautious with this interpretation as our results may be limited by participant recall bias and TBI severity (not assessed in this study). Grouping such a complex time of development in one age group (grade 4 or before, or 0 to 9 years old) may have masked any significant associations of earlier TBIs with current academic performance.

Each reported grade at first (or only) and most recent TBI was significantly associated with the number of times a student sought medical treatment for any physical injury in the last 12 months, although odds were higher for more visits to treat a physical injury and TBIs occurring in higher grades. Seeking medical treatment for a physical injury was also significant for those who only had one TBI, but the odds ratios were higher for those who had more than one TBI. The number of medically treated physical injuries was particularly high for both males and females if they had a TBI in the last 12 months, compared to TBI history during lifetime except the past 12 months. The odds ratios increased for more visits to seek medical help among older students who had their last TBI between grades 9 to 12 (they were between 14 to 18 years old). Under the assumption that increased medical treatment of physical injuries reflects more injuries, our results are consistent with the literature.[23,14,15] Sports-related injuries, which account for most adolescent TBIs (in this sample), are more common in older ages, especially older adolescent males whose size and strength creates greater force and impact in events that may lead to injuries.[41–43] Previous research also indicates that having any previous injury increases an adolescent’s risk for more injuries.[43] It is already known that having one TBI increases one’s risk of having another TBI, but our findings suggest that TBIs, especially those occurring in older adolescence, also increase one’s risk for other physical injury.[23,14,15] To our knowledge this is the first time this association has been documented. Increased physical injuries following a TBI may occur because following a TBI an individual’s ability to safely navigate their environment is significantly affected.[44,45] Future research should consider whether the adverse events associated with TBIs (e.g., substance use) are the main cause in the TBI-related increased risk of physical injuries, or whether the physical impairments brought about by a TBIs (e.g., loss of balance, and vision and auditory acute or chronic impairments) lead to higher risk of physical injuries following TBIs.[11,12,15,18–22,45]

Sports-related TBIs accounted for almost half of the TBIs reported in this study. Sports-related TBIs have consistently been shown to be a major cause of TBIs in adolescents.[2,8,11,37,38,46] Youth and young adults, ages 5 to 24 years, are 8 times as likely to have a sports-related TBI than any other age group when presenting to the emergency department with a TBI, and rates have been increasing.[37,38] Team sports, in particular, rate as main mechanisms for incurring a TBIs among youth.[14] Football, hockey, lacrosse, wrestling, soccer, and basketball (all but one are team sports) have been highlighted as sports with the highest rates of TBIs.[8] Our data reveals that TBIs occurring in hockey were more frequent than TBIs incurred during soccer. This may suggest that sports with more contact (e.g., hockey) present a greater risk for TBIs. While engagement in sports is a positive aspect in the lives of adolescents, due to numerous reports outlining adverse socialization patterns involving substance use and abuse that accompany sport activities, they could also have adverse effects and may lead to more serious or repeated injuries.[2,11,12,47,48] These studies suggest that adolescent athletes may be engaging in harmful behaviors depending on the type of sport they may be practicing. For instance, high contact sports (e.g., football, hockey) may tend to socialize youth to view aggression, pain and risk as normal features in the sporting context that may promote risky behaviors on and off the playing field.[47,48] More efforts should be made to prevent and minimize the risk of TBI in high contact sports.

While motor vehicle collisions (MVCs) have been reported as significant causes of incurring a TBI in adolescence, they made up 5.4% of the reported mechanisms of TBIs in the data we examined in our study.[38,39] Previous OSDUHS results also failed to find MVCs to be a major mechanism of adolescent TBI.[2,11] The discrepancy between our findings and the literature could be due to the different age ranges studied and/or the source of data. The OSDUHS represents a sample of 12 to 18 year-old, whereas previous studies on the mechanism and prevalence of TBIs place adolescents into two groups: 5 to 14 year-old and 15 to 24 year-olds[37–39] In 2014, there were approximately equal proportions of 16 to 20 year-olds involved in MVCs (not specific to TBIs) as 21 to 24 year-old in Ontario, suggesting that most of the MVC-related TBIs occurred at ages above those examined in this study.[41] Furthermore, previous studies examined only medically treated TBIs that presented to the emergency department.[37–39] Future studies should examine if TBIs treated medically are representative of the different mechanisms of TBIs. (e.g., does a higher percentage of MVC-related TBIs present to emergency departments than sports-related TBIs). We found that also falls-related TBIs were another major mechanism of TBIs (19.%). Although not as prevalent as sports-related TBIs among adolescents, fall-related TBIs are the number one cause of TBIs amongst all age groups and account for most of the increase in all TBIs between 2007 to 2013.[39]

Several limitations of the study need to be kept in mind when interpreting the results. Although memory decay is less likely to occur for salient health events, such as TBI (48) raising the possibility of decayed recall of previous TBIs during childhood is a possible limitation. However, there was no evidence of nonresponse bias in reporting on the history of TBI; reports of history of TBI did not differ between classes with response rates above 70% and those with lower rates. Causal inferences cannot be made because of the cross-sectional nature of the study. This study did not assess TBI severity which has been shown to have a dose-dependent impact on adverse outcomes.[24–25,30,49] The prevalence of TBIs reported is still likely an underestimation as the definition used for this study excluded TBIs that did not result in a loss of consciousness, a loss of consciousness that lasted less than 5 minutes, and TBIs that did not require overnight hospitalization. By excluding certain adolescent populations, this study may have missed students who are a higher risk of TBIs (i.e., institutionalized students). TBIs are overrepresented in the incarcerated population, which may have influenced our findings.[50,51] Lastly our analyses were not controlled for students’ socioeconomic status and ethnicity which may further shed light on the reported associations. Future studies should consider measuring and adjusting the analyses for this possible source of bias.

## Conclusion

This study provides evidence that TBIs influence adolescent academic performance and physical health. Although the first (or only) and most recent TBIs were associated with lower academic performance and more frequent visits to treat physical injuries, the most recent TBIs, as well reporting multiple TBIs, had a greater association with the outcomes observed here. The grade at which the TBIs occurred was related to the two observed outcomes. TBIs occurring in grades 5 to 8 had higher and significant odds of poorer academic performance whereas students who reported more visits for medical treatment of physical injuries were more likely to have had TBIs in higher grades. More studies are needed to determine the grades or time of development where TBIs are most strongly associated with both short term and longer term adverse outcomes. TBIs incurred between 15 to 24 years of age are associated with the highest lifetime costs to the individual and society, which are significantly attributed to loss of productivity.[38] Results of the current study suggest that the age at which a TBI occurs is an important factor in the impact on academic performance and number of visits to see a medical professional for physical injuries. These results highlight the importance of injury prevention and public health policies needed to create environments that reduce the risk of TBIs. Healthcare workers, educators and families need to be aware of an adolescent’s TBI history to support them as needed to avoid adverse outcomes.

Keeping these limitations in mind, the results of this study provide important information on sport participation and TBI, incurred physical injuries treated by a medical professional and influence on academic performance that can help inform TBI prevention and intervention strategies. While progress is occurring regarding diagnosis, treatment protocols and education little is known about how to translate this new knowledge to stakeholders. Given the prevalence of sports concussion, and the importance that recognition of concussion symptoms in preventing repeated concussions and second impact syndrome, it is particularly salient that relevant stakeholders such as athletes, parents, teachers, coaches, school administrative staff, healthcare providers, researchers, government agencies have relevant and accurate information. Given the frequency of concussion in society and the potential personal and societal burden, strategies for the prevention of concussion are urgently required.
